# Indicators to Assess Physical Health of Children and Adolescents in Activity Research—A Scoping Review

**DOI:** 10.3390/ijerph182010711

**Published:** 2021-10-13

**Authors:** Simon Kolb, Alexander Burchartz, Doris Oriwol, Steffen C. E. Schmidt, Alexander Woll, Claudia Niessner

**Affiliations:** 1 Institute of Sports and Sport Science, Karlsruhe Institute of Technology, 76131 Karlsruhe, Germany; alexander.burchartz@kit.edu (A.B.); steffen.schmidt@kit.edu (S.C.E.S.); alexander.woll@kit.edu (A.W.); claudia.niessner@kit.edu (C.N.); 2 Institute of Movement and Sport, University of Education Karlsruhe, 76133 Karlsruhe, Germany; doris.oriwol@ph-karlsruhe.de

**Keywords:** body composition, cardiometabolic biomarkers, physical fitness, youth, physical activity, sedentary behavior

## Abstract

Sufficient physical activity can help promote and maintain health, while its lack can jeopardize it. Since health and physical activity lay their foundation for later life in childhood and adolescence, it is important to examine this relationship from the beginning. Therefore, this scoping review aims to provide an overview of physical health indicators in children and adolescents in research on the effects of physical activity and sedentary behavior. We identified the indicators used to quantify or assess physical health and summarized the methods used to measure these indicators. We systematically searched Scopus, Pubmed, and Web of Science databases for systematic reviews. The search yielded 4595 records from which 32 records were included in the review. The measurements for physical health reported in the reviews contained measures of body composition, cardiometabolic biomarkers, physical fitness, harm/injury, or bone health. Body composition was the most used indicator to assess and evaluate physical health in children, whereas information on harm and injury was barely available. In future research longitudinal studies are mandatory to focus on the prospective relationships between physical activity or sedentary behavior, and physical health.

## 1. Introduction

Physical inactivity is one of the leading risk factors for all-cause mortality reported by the World Health Organization (WHO) [1]. Four of the other top 10 factors are related to physical activity (PA) as well. Together those five factors (high blood pressure, high blood glucose, overweight and obesity, physical inactivity, and high blood cholesterol) were accountable for 19.5 million deaths worldwide in 2004 [1].

The leading risk factor is hypertension and is the most common risk factor for cardio-vascular diseases (CVD). Strong evidence demonstrates that PA reduces blood pressure (BP) among adults with normal BP, prehypertension, and hypertension, which makes PA suitable to prevent hypertension as well as treat it [2]. Additionally, PA seems to be appropriate to reduce high cholesterol. Poitras et al. [3] reported favorable associations of total PA with total cholesterol and MVPA with non-HDL cholesterol.

Moreover, PA could be an effective way to reduce blood glucose levels: A theoretical approach by Hansen et al. [4] showed that reallocating time from sedentary behavior (SED) to moderate-to-vigorous physical activity (MVPA) would lead to reduced glucose levels in adolescents. In addition, PA interventions showed effectiveness in reducing HbA1c levels in non-diabetic populations [5]. With clustered cardiometabolic risk showing stability from childhood to adulthood [6], monitoring and prevention needs to start early.

Overweight and obesity are also not only a leading risk factor in the adult population. As overweight and obesity in childhood are associated with a greater risk of chronic diseases such as type 2 diabetes in later life [7], they are already a problem in children. While it is difficult to lose weight or to maintain healthy weight after weight loss [8], it is important to prevent excessive weight gain in childhood and adolescents. Data from the International Children’s Accelerometer Database (ICAD) showed associations between MVPA and lower body mass index (BMI) and waist circumference (WC) z-scores. Those associations were stronger at the higher percentiles, suggesting that increasing MVPA could help to lower the number of children and adolescents at the upper tail of BMI and WC frequency distributions [9]. Considering that overweight and obese children often become overweight and obese adults [10], early countermeasures are necessary.

The lack of physical activity in school-aged children is already associated with adverse physical health outcomes and reduced physical fitness [3,11]. On top of that, PA in childhood seems to predict PA levels in later life [12,13]. As mentioned above, clustered cardiometabolic risk showed stability from childhood into adulthood [6] and similar findings are available for obesity [10]. This suggests that health status during childhood directly affects the adult health status.

Against this background, it should be in the general interest to educate healthy and active children who will grow into healthy and active adults to tackle those issues.

Therefore, this scoping review aimed to provide an overview of physical health indicators in children and adolescents in research on the effects of PA and SED. The aim was to identify the indicators used to quantify or assess physical health and summarize the methods used to record these indicators. This review is intended to serve as a basis for planning studies for future research, thus contributing to further analysis of the effects of PA and SED on physical health of children and adolescents.

## 2. Materials and Methods

Although, we conducted a scoping study, we adopted the methods of a systematic review regarding literature search, inclusion/exclusion of literature, and data collection as proposed in the PRISMA statement [14]. Eligibility criteria were defined a priori to ensure consistency in decision-making.

### 2.1. Eligibility Criteria

The PICOS framework [15] was used as an orientation to define eligibility criteria and to ease the searching process. We used the following criteria for the inclusion:

(a) To identify the most important indicators used in the field, the scoping review focused on systematic reviews (SR) and meta-analyses; (b) We evaluated SRs on healthy children and adolescents aged 6 to 17 years. To get a preferably realistic result, participants with special conditions (e.g., disabilities or chronic diseases) were no reason for exclusion as long as they were part of cross-sectional samples. Whereas studies entirely focusing on participants with special conditions were excluded; (c) All forms of PA or lack of PA with reported effects on physical health were considered. Sedentary behavior (SED) is defined as waking behavior with an energy expenditure equal to or less than 1.5 metabolic equivalents (METs) [16]. Therefore, it can be judged as a form of activity with low intensity, and SRs conducted on SED as exposure were considered eligible; (d) A control group was not applicable due to the focus on SRs; and (e) As this scoping review aims to identify the indicators to assess physical health, the included SRs must at least include one aspect of physical health.

### 2.2. Information Sources and Search

PubMed (1946–May 2020), Scopus (1960–May 2020), and Web of Sciences (Core Collection, 1945–May 2020) were used to identify relevant articles. Additionally, the Cochrane Library was checked for relevant entries. The references of the records were checked as well for additional relevant citations. The search terms consisted of variations and combinations of the keywords “physical activity”, “children”, and “health”. The full search is available upon request. The search was limited to results published in English or German.

### 2.3. Study Selection

Citations were downloaded into a reference management software (Citavi 6.5, Swiss Academic Software GmbH, Wädenswil, Switzerland) and duplicates were removed automatically. Two reviewers (S.K., A.B.) screened the title and abstract of the remaining references. An agreement was essential for exclusion at this level. Full texts were then screened against the eligibility criteria by two reviewers (S.K., A.B.) and agreement was required for inclusion in the review. If agreement at any level could not be reached through discussion a third reviewer (C.N.) was contacted and a decision was made by majority vote.

### 2.4. Data Collection and Items

The data-extraction form was created by the first author and reviewed by the other authors. Each record was assigned a unique identifier. Extraction was completed in Excel (Microsoft Corp., Redmond, WA, USA) by one reviewer (S.K.) and checked for accuracy by another (A.B.). Characteristics of the SR (author, title, year of publication, number of studies in the SR/MA, an age range covered), characteristics of the PA or SED measurement, information if a meta-analysis was conducted with several included studies, and health indicators, as well as measurements used to assess those indicators, were extracted. Reviewers were not blinded to the authors or journals when extracting data. The data-extraction form is available upon request by the corresponding author.

### 2.5. Synthesis of Results

The results were grouped by the reported exposure and structured around the reported health indicators representing physical health and are presented as a narrative synthesis.

## 3. Results

### 3.1. Electronic Search

**Figure 1:**
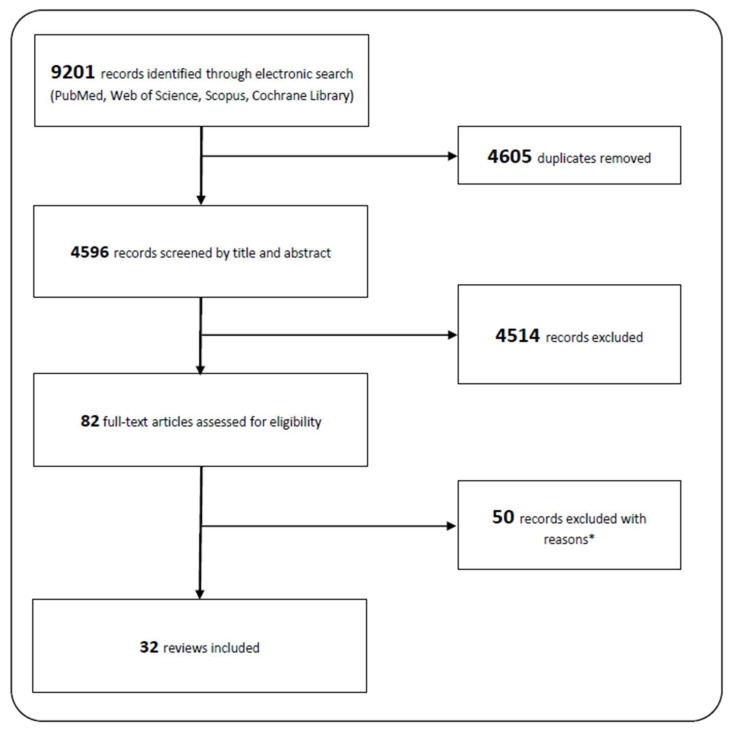
Flow-chart literature search and study selection process * Reasons for exclusion: no systematic review (e.g., conference paper, mini review…; 26), no physical health indicator (4), no PA or SED measure (4), special population (2), wrong age (4), missing information (3), and wrong focus, e.g., comparison of training effects (7).

The systematic literature search, as presented in Figure 1, was conducted according to the PRISMA guidelines. The search yielded 9201 records. After the removal of duplicates, 4595 records remained and 4513 were excluded after screening titles and abstracts. The remaining 82 records were assessed for eligibility and 50 records were excluded with reasons. A full list of the reasons for exclusion is available upon request. Thus, 32 records remain for inclusion in the analysis.

### 3.2. Study Characteristics

According to the inclusion criteria, systematic reviews and meta-analysis were suitable for inclusion. Since this scoping review is aimed at the indicators used, it is of secondary importance whether the source is a systematic review or a meta-analysis. Therefore, to enhance the readability of the manuscript SR refers to both in the further course.

Table 1, Table 2 and Table 3 present the characteristics of the 32 SR stratified by the exposure of the included studies. Seventeen of the identified reviews focused on PA as exposure, eleven focused on SED, and four SRs included studies focused on PA as well as studies focused on SED.

The number of included studies for the SRs ranged from eight to 235. Twenty-one SR included ten to 50 studies, three SR included less than ten studies, three SR included 50 to 100 studies, and four SR included more than 100 studies.

### 3.3. Health Indicators

The measurements reported in the SRs could be summarized as measurements of body composition (BC), cardiometabolic biomarkers (CMB), physical fitness (PF), harms/injuries (H&I), or bone health (BH).

#### 3.3.1. SR with PA as Exposure

Seventeen SR, published between November 2010 and April 2020, evaluating the effects of PA on the physical health of children and adolescents were identified by our search.

- Body Composition

Ten SR included body composition as an indicator for physical health. Body composition was used as an indicator for physical health in association with active travel/commuting [21,23,27], active video games [17], pedometer-determined [24], or objective measured PA [3], and PA with no further specifications [20,25,26,30].

Body mass index (BMI), as well as thickness of skinfolds (SF) or sum of skinfolds (SSF) were reported in at least one study in each of the 10 SR or MA. The second most common variable to assess body composition was waist circumference (WC) which was presented in 8/10 SR or MA. Other measurements used to assess and evaluate body composition reported by the studies in the SR were body-fat percentage (%BF) (6/10), fat mass (FM) (3/10), fat mass index (FMI) (2/10), fat-free mass (FFM) (2/10), and fat-free mass index (FFMI) (2/10).

One SR was conducted on prospective studies [30] and body-weight change or body-fat percentage change were used in at least one study to evaluate body composition.

- Cardiometabolic Biomarkers

Cardiometabolic biomarkers were used as an indicator for physical health in four SR. They were investigated in association with active video games [17], active travel [27], objective measured PA [3], and PA with no further specifications [20].

All 4 SR including studies reporting on blood lipids as well as such reporting on insulin resistance or sensitivity. The measured values for the assessment of blood lipid levels included the following: total cholesterol, HDL cholesterol, triglycerides, and more. The homeostasis model of assessment of insulin resistance (HOMA-IR), fasting insulin levels, fasting glucose levels, among others, were used to assess insulin resistance or sensitivity.

Moreover, different blood pressure measurements, inflammatory markers, or properties and functions of the arterial system were used to assess cardiometabolic health. Some of the included studies also used clustered risk factors or metabolic syndrome scores to evaluate cardiometabolic health.

- Physical Fitness

Five SR used physical fitness as an indicator for physical health. Physical fitness was used in association with active travel respectively commuting [21,23,27], active video games [17], and objectively measured PA [3].

All five SR report on cardiorespiratory fitness. 4/5 SR report on muscular fitness and flexibility. One SR reports on balance and one SR includes at least one study on agility and speed.

- Harms and Injury

Harms and injuries were only addressed in one SR [28] focused on neck and low back pain. Pain was assessed via questionnaire.

- Bone Health

Bone health was used as an indicator for physical health in seven SR and evaluated in association with objectively measured PA [3], PA without further specifications [22,29,31,32], and swimming [18,19].

Bone mineral density (BMD) was reported by all seven SR. The second most common variable to assess bone health was bone mineral content which was reported in 6/7 SR.

Other metrics used to measure bone health include bone age, total skeletal area, bone stress index, bone strength index, strength–strain index, cross-sectional moment of inertia, cortical thickness, or polar moment of inertia.

#### 3.3.2. SR with SED as Exposure

Eleven SR published between March 2008 and October 2019 on the effects of SED on the physical health of children and adolescents were identified by our search. None of them reporting on harm and injury as an indicator for physical health.

- Body Composition

In SR using SED as the exposure body composition was used as an indicator for physical health in 9/11 [33,34,35,36,39,40,41,42,43] identified SR conducted between 2008 and 2019.

BMI was the most common method to assess body composition reported in each of the nine SR, followed by WC (8/9), body-fat percentage (6/9), and skinfold thickness respectively sum of skinfolds (6/9). Other metrics to assess and evaluate body composition reported by at least one of the studies included in the SR were fat mass/body fat (5/9), weight-to-height ratio (WHtR) (4/9), fat mass index (2/9), fat-free mass/lean body mass (1/9), lean body mass percentage (1/9), hip circumference (1/9), and waist-to-height ratio (1/9).

- Cardiometabolic Biomarkers

Seven of the eleven SR [33,34,36,38,40,42,43] used cardiometabolic biomarkers as an indicator for physical health.

Blood pressure was reported in all seven SR as an indicator of cardiometabolic health. Blood lipid scores, including triglycerides, total cholesterol, or HDL cholesterol among others, were reported in five of the seven SR or MA. Measurements of insulin sensitivity respectively resistance or glucose were also reported by five of the SR. Three of the SR include studies using a combination of cardiometabolic risk factors, clustered cardio-metabolic risk, or risk of metabolic syndrome to evaluate cardiometabolic health.

- Physical Fitness

Physical fitness was used as an indicator for physical health in four of the eleven SR on SED [33,34,42,43]. All four SR include studies on cardiorespiratory fitness or strength/muscular fitness. In addition, one SR [33] included studies on flexibility and power.

- Bone Health

Three of eleven SR on the effects of SED in children and adolescents included studies on bone health [34,37,43].

Two of those SR [34,43] only report on bone mass as a measurement of bone health. The metrics used in the studies of the third SR [37] include BMD, BMC, bone architecture, bone strength, stiffness index, and speed of sound as measurements of bone health.

#### 3.3.3. SR with PA and SED as Exposure

Our search yielded four results for SR on physical health aspects of children or adolescents that included studies with PA as well as SED as exposure in their analysis. The four SR were published between May 2012 and January 2019. All of them focused on either body composition, cardiometabolic biomarkers, or both as a health indicator.

- Body Composition

One SR solely reports on the effects of PA or SED on body composition while two SR report on body composition and cardiometabolic biomarkers.

BMI and WC were reported by studies of all three SR. 2/3 also report on SF. One SR each report on body-fat percentage and body-fat mass.

- Cardiometabolic Biomarkers

One SR was conducted on the effects of PA or SED on the metabolic syndrome, while two SR report results for cardiometabolic biomarkers and body composition.

The one SR on the effects of PA or SED on metabolic syndrome only includes studies reporting on clustered risk factors according to the definition of metabolic syndrome.

The two other SR reporting on cardiometabolic biomarkers include studies on blood lipids, blood pressure, or insulin sensitivity resp. resistance. One of them also includes at least one study reporting results for inflammatory markers.

## 4. Discussion

This scoping review aimed to provide an overview of physical health indicators in children and adolescents in research on the effects of PA and SED. The aim was to identify the indicators used to quantify or assess physical health and summarize the methods used to record these indicators. We identified body composition, cardiometabolic biomarkers, physical fitness, harm and injury, and bone health as the relevant indicators to assess physical health in research on effects of physical activity or sedentary behavior in school-aged children. These indicators match those proposed within the framework of the evidence update for the Canadian 24 h Movement Guidelines [3]. The current search did not reveal further indicators for physical health.

Body composition was analyzed in 22/32 SR. The ability to derive measures of body composition, such as BMI, from easy-to-assess anthropometric variables is possibly one reason for the high prevalence of this health indicator. The most common proxy variable to assess BC was BMI. BMI calculated from objectively assessed height and weight, as well as BMI calculated based on self or proxy reported data, were used. BMI for age or BMI-z-scores was often used to account for age and sex of the participants. The BMI is often used to divide subjects into groups, e.g., underweight, normal weight, or overweight. It is also used to classify levels of adiposity. Using BMI as a health indicator, some limitations need to be considered. Although being used to measure body fat, BMI is an indirect measurement and it does not account for age, sex, bone structure, fat distribution, or muscle mass [48]. Therefore, it is recommended to use BMI for age to account for sex and age, which is important when analyzing children [49]. A meta-analysis of the diagnostic performance of BMI to identify obesity showed high specificity, but moderate sensitivity in children and adolescents [50]. Lacking sensitivity leads to misclassification of obese children as non-obese, which leads to missed opportunities for intervention. Moreover, as an indirect measurement, BMI is not a precise predictor of body fat. Assuming a BMI score of 20 kg/m^2^, the corresponding body-fat percentage value can range from 5 to 40% [51]. Looked at the other way around, it can be seen that with a body-fat percentage of 20%, the corresponding BMI can be between 15 and 30 kg/m^2^ [51]. Nevertheless, there is consistent evidence that a high BMI for age is an accurate measure for a high body-fat content and it could serve as appropriate tool to diagnose overweight and obesity in children [49]. Therefore, the use of the BMI for age should be seriously considered when examining children.

The second most commonly used method for assessing BC was the measurement of skinfold thickness at one or more predefined locations. Subsequently, the body-fat percentage is calculated by means of a formula, such as the one developed by Slaughter et al. [52]. While skinfolds show a stronger association with body fat than BMI [53], there are some limitations to be considered as well. A study by Kriemler et al. showed that for subjects over the 95th BMI-percentile the skinfolds do not add to the prediction of body fat or the prognostic value of BMI [54]. Moreover, the measurement of skinfolds is error-prone and highly dependent on the observer [55].

Cardiometabolic biomarkers were used in 14/32 SR and to assess physical health. Blood lipid levels are reported in all 14 SR and measures of blood pressure in 13/14 SR. Reported blood lipid levels mostly include triglycerides, total cholesterol, or HDL cholesterol. In addition, there are various ratios of the individual values (e.g., HDL/TC) or additional values such as LDL cholesterol. Laboratory analysis is necessary to determine the blood lipid levels. With laboratory analysis usually leading to higher costs, this can be an explanation why they are not as often used as measurements of body composition in studies with large samples.

Systolic blood pressure, diastolic blood pressure, and mean arterial blood pressure are reported as measurements of blood pressure. Blood pressure is often used to classify the subjects into subjects with hypertension and those without. Auscultatory measurement of BP is recommended by current guidelines for the diagnosis of hypertension in children. This is mainly caused by the fact that available thresholds have been obtained by the auscultatory method. Additionally, BP measurements by auscultatory method and automated electronic method are not necessarily interchangeable [56]. Considering that interchangeability is not necessarily given between BP measurements, caution is needed when analyzing results from different sources.

Physical fitness is expressed through cardiorespiratory or aerobic fitness in all nine SR. Nevertheless, the evaluation is based on different methods including VO2max levels or performance in 6 min runs or shuttle run tests. Other proxy variables were muscular fitness (3/9), strength (5/9), or flexibility (4/9). Single studies also used speed or agility as a proxy for PF. In the field of physical fitness research, the inconsistency in the recording of PF is a well-known limitation [57]. Furthermore, the parameter “physical fitness” as health outcome must be considered cautiously, since studies consider PF to be an independent factor affecting health, rather than a part of health [58].

The limited information on the association of PA and harms or injury is striking, but in line with other findings: Poitras et al. [3] could not identify any study on injuries and harms in their review for the update of the Canadian Physical Activity Guidelines for Children and Youth. Although, harms were judged a critical health indicator by their expert panel. Others highlighted the limited number of studies available for health indicators other than anthropometrics as a limitation [43].

Bone health was used as an indicator in ten SR, seven of which exclusively focused on bone health. A possible explanation may lie within the assessment of the variables for bone health. Specific instruments are needed such as X-ray machines, or ultrasonic devices and their measurements are independent of other variables such as anthropometrics.

Looking at the variables used as proxies for the health indicators, it is striking that on the one hand, there are numerous different variables for the same indicator, e.g., BMI, skinfold measures, body-fat percentage, or fat-free mass to assess body composition. On the other hand, we recognized some widespread proxy for the health indicators used in a broad range of SR and their included studies. The most common proxy measures reported in the reviews were BMI for body composition, blood pressure for cardiometabolic biomarkers, cardiorespiratory/aerobic fitness for physical fitness, and bone mineral content for bone health. Even though those proxy measures are frequently used and reported by numerous studies, it is mandatory to consider the assessment method and protocol to compare the results and effects of different investigations.

Considering the reported relationships between PA and physical health in the reviews, the overall picture supports a positive association between PA and body composition (BC), cardiometabolic biomarkers (CMB), and physical fitness (PF).

Favorable effects on BC were reported for total PA [3], active travel [23,27], and higher levels of walking [24]. In contrast, two SR found whether consistent evidence for a prospective association of BC and MVPA [46] nor an association between objectively measured activity patterns and BC [47]. Other aspects like nutrition respectively energy intake could be a possible explanation for the inconsistent prospective association of MVPA and BC. Verswijveren et al. (2018) report inconsistent evidence, which may be caused by methodological differences. They used summative coding with an a priori criteria to be coded as evidence and report that many analyzed activity patterns did not meet the criteria [47]. This also applies to the inconsistent effects on CMB and PF found by their review.

One SR [21] reports ambiguous results for the effect of active travel on PF. This conflicts with the findings of two other SRs [23,27]. While most studies found a positive relationship between active travel and PF, the authors felt the need to identify potential influencing factors or mediators to avoid misconceptions. Therefore, they judged the evidence as inconsistent [21].

As mentioned before, there is a paucity of reviews analyzing harms or injuries as a health indicator. The only SR included in this scoping review had to deal with the limited available evidence.

Due to different research foci, the evaluation of different forms of PA, and the combination of PA with other factors, bone-health outcomes are not comparable across the evaluated SR.

The overall picture regarding the relationships between SED and the indicators for physical health is less consistent and clear compared to PA. A reason could be differences in the assessment of sedentary behavior compared to PA. Common proxy variables for SED are TV viewing or total sedentary time. Using TV viewing as a proxy measure, it is obvious that sitting on a couch and solely watching TV and watching TV while eating some snacks or consuming a sweetened drink or alcohol most likely have different effects on health. Therefore, differences in dietary behavior associated with TV viewing could explain observed differences in various studies. Another problem arises when focusing on the total amount of sedentary time as a proxy. Conflicting results between studies or between participants within a study may be caused by behavioral differences during the non-sedentary periods. A study by Ekelund et al. [59] found higher levels of MVPA favorable for cardiometabolic risk factors independent of time spent sedentary.

We recommend that when conducting studies of the effect of physical activity or sedentary behavior on physical health in children and adolescents, the choice of survey methods for assessing physical health indicators should be critically considered. Even if the cost and effort of the method are a critical point, especially for large studies, reproducibility, error-proneness, and informativeness are important factors that have to be considered when selecting the method to assess physical health parameters.

Furthermore, given the different dimensions of health (e.g., physical health, mental health, social health, etc.), we recommend that authors clarify which aspects of health they are addressing in their work. During the literature search for this review, we encountered several articles in which neither the title nor the abstract gave any indication of the aspect of health being studied. This scoping review has some limitations that need to be mentioned. Considering only indicators related to physical health, the reported indicators also do not represent a holistic understanding of health. For a comprehensive examination of the health status, additional information, e.g., on psychological and social aspects, is needed.

Since only published papers were included in the review, publication bias may be a case.

A strength of this scoping review is the inclusion of PA as well as the lack of PA as exposure. Thus, not only were the health indicators evaluated for which positive effects from PA could be expected, but also those with negative effects from lack of PA. As a result, this scoping review gives an overview of the indicators used to evaluate the effects of PA or lack of PA on physical health in children and adolescents and could serve as guidance for upcoming studies on physical health and PA.

Additionally, the studies included in SR have usually already passed a quality assessment, lowering the risk of biased information.

## 5. Conclusions

The physical aspects of health in children and adolescents are typically assessed through indicators of body composition, cardiometabolic health, physical fitness, and bone health. Depending on the aspect to be investigated, it is advisable either to combine one exposure (e.g., PA) with several indicators or to investigate the influence of different behavioral patterns on one indicator. Overall, measures of PA show higher associations to health indicators compared to measures of SED among youth.

Further research is needed on possible negative consequences of physical activity or inactivity such as injury and harm, as well as on possible prevention of harm or pain through PA.

While cross-sectional associations are frequently investigated, more long-term studies are needed to analyze the prospective relationships between physical activity or its lack and the different health indicators. Future research needs to focus on potential influencing factors on those relationships to gain a deeper understanding of how physical activity and sedentary behavior affect the health of children and adolescents.

## Figures and Tables

**Table 1:** Characteristics of the included reviews including studies on effects of PA.

Reference	# Studies	Exposure	Outcome
Gao et al., 2015, [17]	35	Active video games	Health outcomes
Gomez-Bruton et al., 2013, [18]	23	Swimming	Bone tissue
Gomez–Bruton et al., 2016, [19]	14	Swimming	Bone mineral density
Guinhouya et al., 2011, [20]	37	PA	Metabolic syndrome, insulin resistance
Henriques-Neto et al., 2020, [21]	11	Active commuting	Physical fitness
Krahnebühl et al., 2018, [22]	21	PA	Bone geometry
Lubans et al., 2011, [23]	27	Active travel	Health-related fitness
Miguel-Berges et al., 2017, [24]	36	PA	Adiposity
Poitras et al., 2016, [3]	162	PA	Health indicators
Ramires et al., 2015, [25]	18	PA, inactivity	Body fat, obesity
Rauner et al., 2013, [26]	14	PA, PF	Overweight
Saunders et al., 2013, [27]	8	Active travel	Health benefits
Sitthipornvorakul et al., 2010, [28]	13	PA	Neck pain, low back pain
Tan et al., 2014, [29]	37	PA	Bone strength
Wilks et al., 2011, [30]	14	PA	Obesity
Yang et al., 2020, [31]	9	PA, calcium	Bone health
Zulfarina et al., 2016, [32]	9	PA	Bone mineral acquisition

#—Number of included studies.

**Table 2:** Characteristics of the included reviews including studies on effects of SED.

Reference	# Studies	Exposure	Outcome
Carson et al., 2016, [33]	235	SED	Health indicators
Chinapaw et al., 2011, [34]	31	SED	Biomedical health indicators
Cliff et al., 2016, [35]	88	SED	Health and development
Fröberg and Raustorp, 2014, [36]	45	SED	Cardio-metabolic risk
Koedijk et al., 2014, [37]	17	SED	Bone health
Lee and Wong, 2015, [38]	24	SED	Blood pressure
Rey-Lopez et al., 2008, [39]	71	SED	Obesity
Ribeiro Canabrava et al., 2019, [40]	50	SED	Cardiovascular risk
Tanaka et al., 2014, [41]	13	SED	Adiposity
Tremblay et al., 2011, [42]	232	SED	Health indicators
Van Ekris et al., 2016, [43]	109	SED	Biomedical health indicators

#—Number of included studies.

**Table 3:** Characteristics of the included reviews including studies on effects of SED and PA.

Reference	# Studies	Exposure	Outcome
Oliveira and Guedes, 2016, [44]	18	PA, SED	CRF, Metabolic Syndrome
Prentice-Dunn et al., 2012, [45]	17	PA, SED	Childhood obesity
Skrede et al., 2018, [46]	30	SED, MVPA	Cardio–metabolic risk factors
Verswijveren et al., 2018, [47]	29	Activity patterns	Cardio–metabolic risk factors

#—Number of included studies.

## Data Availability

Search terms, data extraction form, and a list of excluded references are available upon request by the corresponding author.
